# A Text from a Sermon

**Published:** 1892-01

**Authors:** 


### A TEXT FROM A SERMON.

On Sunday, December 6, 1891, that eminent and famous Baptist divine, the Rev. J. B. Hawthorne, D. D., preached an able sermon upon the subject of "*Lies and Liars.*" We take as *our* text the following paragraph, published in an abstract of the sermon in the *Atlanta Constitution* of the following day :

" *In this category I would place the blood purifier and the liver-pad man, who have discovered the greatest remedies of the age.*"

"This category" is the category of *liars*. Dr. Hawthorne always preaches on timely subjects and is noted for the fearlessness with which he handles them, no matter whose feelings may be hurt. This fearlessness is the more commendable when we remember that it is not conducive to the preacher's personal popularity. The "blood purifier man" keeps pace with medical progress—as may be seen from his advertisements. If disease is due to "*a humor in the blood*," his medicine is the "purifier" which cleanses the system of the offending substance. If the disease is due to "*germs*," he cries, presto! change, and straightway the advertisement tells the afflicted people that the medicine *kills* these *germs*, or "*drives them out through the pores*."

Now we get to the point desired, and are able to relieve the doctor of our first suspicion that his condemnation of "blood purifier and liver-pad men" did not cover the ground, and of the suspicion that "germ-killer" men were not to be classified with the "blood-purifier" liars, for they are now all in the same "category."

Two patent medicines will be remembered as having been extensively advertised a year ago, and one of these was editorially commented upon by one of the city papers—this editorial being commendatory, and, being in that part of the paper where the reader seeks editorial brain pabulum, was duly absorbed and digested by both laity and doctors.

These "germ-killers" were known respectively as "Microbe Killer" and "*Royal Germateur*." The former was a Texas product, and we will dismiss it to the consideration of its fellow citizens. The latter is (to the writer) of uncertain origin, but the ownership, alas, of some of the stock of this company has been said to be vested in certain *Georgia preachers*, and not any of your "cross-roads," "one-horse" or local preachers, but preachers whose signature to the certificates of the worth of "*Germateur*" would exert a national influence. Now, let us not be understood as *saying* that a preacher who privately *owned*

*stock* in a patent medicine would try to increase *its* sales, and *his* dividends, by allowing his name to appear at the bottom (or top, in large letters) of a certificate as to the "medicine's" curative properties. That would not be in good taste. The bad blood-purifier (or germ-expeller, not germ-*killer*) man might do such a thing.

Now we come back to the puzzling question of how much "blood-purifier man" covers in the sermon? Is a "blood-purifier man" supposed to be a "bigger," or the only, liar; or, as the "blood-purifier man" *runs the germs out*, does that qualify him as a liar, or must we let the blood-purifier, the germ-expeller and the germ-killer man come under the condemnation of our text?

We are constrained to believe that we must, and to commend our eminent preacher for the candor and fearlessness of his characterization, and for his daring to do and speak "right" even if in so doing his "brother" ministers fall under the ban. We hope the words of the sermon may influence said ministers to sell their stock, or give it away, *if they still own it*, and thus stand aside from the "category of *liars*," and array themselves, *purged and forgiven of lies*, on the side of *decency*, *consistency* and *truth*.

"Germateur" is said to be in a bad way; large advertisements no longer appear, and the *expose* of its contents has doubtless rendered it no longer a profitable investment. But even if it *is*, what influence can that exert upon the *preachers* who stand condemned by the burning words of our text?

"LIES AND LIARS."

"*In this category I would place the blood-purifier and the liver-pad man, who have discovered the greatest remedies of the age.*"—Rev. J. B. Hawthorne, Sunday, Dec. 6, 1891; *Constitution* report Monday, Dec. 7, 1891.

